# How pubertal timing and self-regulation predict adolescent sexual activity in resource-poor environments

**DOI:** 10.1017/S095457942300127X

**Published:** 2023-10-04

**Authors:** Roy Otten, Thao Ha, Erika Westling, Kathryn Lemery-Chalfant, Melvin N. Wilson, Daniel S. Shaw

**Affiliations:** 1 Department of Psychology, Radboud University Nijmegen, Nijmegen, NL, USA; 2 Department of Psychology, Arizona State University, Tempe, AZ, USA; 3 Oregon Research Institute, Springfield, OR, USA; 4 Department of Psychology, University of Virginia, Charlottesville, VA, USA; 5 Department of Psychology, University of Pittsburgh, Pittsburgh, PA, USA

**Keywords:** adolescence, puberty, self-regulation, sexual activity

## Abstract

Studies found support for a link between pubertal timing and self-regulation in low-resource environments. This link could potentially explain a link between pubertal timing and early risk behavior. This study builds on this body of research by examining the mediated effect of pubertal timing on sexual activity through self-regulation in 728 adolescents and their families in a group with poor resources and a group with adequate resources. Income-to-Needs (ITN) was measured at age 7.5 to establish two groups (low-ITN and Medium/High-ITN). Pubertal timing was measured at age 10.5, self-regulation was assessed at age 14 and operationalized with effortful control, and sexual activity was assessed at age 16. Structural equation modeling was employed to test the hypothesized model in both groups. The link between pubertal timing and sexual activity mediated by effortful control was only significant in the low-ITN group. Specifically, more advanced pubertal maturity was associated with lower levels of adolescents’ effortful control, which in turn was associated with more sexual activity at age 16. Findings were partially replicated with a drug use index replacing sexual activity. This study shows a different operating link from pubertal timing to effortful control and subsequent risk behavior in resource-poor environments. Implications are discussed.

## Introduction

### Adolescence and risk behavior

Following the dual-systems model of adolescent risk-taking, adolescence marks a time of rapid development in which physical, cognitive, and behavioral development may not progress at the same rate (Crone & Dahl, 2012; Lambert et al., 2014; Steinberg, 2005). While lack of impulse control, sensation seeking, and reward-seeking can be part of normative adolescent development, these attributes are often exacerbated in those with earlier pubertal development compared to their peers (Steinberg, 2005). Earlier pubertal timing creates a gap between advanced physical development and adolescent’s cognitive abilities to regulate affect and behavior (Steinberg, 2005), underlying adolescents’ engagement in risky behaviors such as unprotected sex and substance use (Casey et al., 2008; Ernst et al., 2005; Steinberg, 2005; Westling et al., 2012).

Engagement in risky behaviors is disproportionally present in adolescents from low-income families. In 2019, among all children under 18 years in the US, 38% lived in low-income families (United States Census Bureau, 2019). Growing up in a low-income family is associated with cognitive and social-behavioral development and physical health. Studies have repeatedly shown higher levels of substance use (Santiago et al., 2013) and sexual risk behaviors (e.g., Langille et al., 2005) in adolescents from low-income families. Specifically, adolescents from low-income families are more likely than adolescents from high-income families to be sexually active (Kipping et al., 2015) and often more likely to engage in frequent sexual intercourse, sometimes with negative health consequences such as unwanted pregnancy and sexually transmitted infections (Langille et al., 2005).

A prominent theoretical framework that aims to explain increased risk behavior in adolescents from low-income environments is Life-History Theory (LHT, Ellis et al., 2012). This evolutionary model posits that stable differences in the environment lead to accelerations in the development of biologically influenced systems of reproduction and behavior, such as pubertal development, in ways that accelerate reproductive fitness that increases the likelihood of offspring earlier in life (Ellis et al., 2012; Dishion et al., 2012). The reproduction (*r*)/Kapazitätsgrenzen (*K*, German for capacity limit)-selection theory is incorporated within the LHT. It states that one’s ecological niche leads individuals either (a) toward earlier pubertal maturation, with more offspring at earlier ages, lower parental investment, and earlier mortality (an *r*-selection strategy), or (b) toward later pubertal maturation, with the production of fewer and later-arriving offspring, higher parental investment, and later mortality (a *K*-selection strategy; Charles & Egan, 2005; Dishion et al., 2012).

Studies have shown that cumulative exposure to a resource-poor environment predicts early pubertal timing in both boys and girls (e.g., Sun et al., 2017). Children growing up in low-income families are more at risk for cumulative stress and insecurity, including parental psychopathology, family conflict, family structure (e.g., Moffitt et al., 1992), and/or abuse and adversity (Belsky et al., 2007; Brooks-Gunn et al., 1985; Ellis & Garber, 2000; Repetti et al., 2002). This experience of stress, in conjunction with a low income, signals a scarcity of resources and instability in the environment. The LHT suggests that this scarcity and uncertainty is responsible for the adaptive biological development for which youth reach pubertal maturation and age of reproduction earlier, and that ultimately increases genetic fitness via a higher likelihood of passing on their genes earlier in life (Belsky et al., 1991).

One mechanistic link relating early pubertal timing with sexual activity is posited to be the gap between physical development and cognitive development, specifically regarding the inhibitory control of impulses, a component of self-regulation. In line with this reasoning, Deater-Deckard et al. (2019) found partial support for this link in their study of 157 Appalachian adolescents. Among youth from poor environments only, more advanced pubertal timing was associated with lower inhibitory control/less effective self-regulation indexed by activation in the prefrontal cortex but not performance during the multisource inference task, assessed via functional MRI (Deater-Deckard et al., 2019).

The present study extends Deater-Deckard et al. (2019) findings by including sexual activity to test links between pubertal timing, self-regulation, and subsequent sexual activity in an environment with poor resources and an environment with adequate resources. We use a sample of ethnically diverse and predominantly lower-income adolescents in a longitudinal design. We hypothesized that the link between pubertal timing, self-regulation, and subsequent sexual activity operates differently in resource-poor environments compared with environments containing adequate resources. Specifically, and in line with postulated mechanisms of LHT and the *r*/K-selection theory, we expected that in resource-poor environments, earlier pubertal timing at age 10.5 would be associated with lower self-regulation at age 14, which in turn would be associated with a higher frequency of sexual activity at age 16. We expect that the underlying mechanisms are similar for substance use, in which adolescents’ advanced puberty and subsequent lower levels of self-regulation will lead to higher substance use (Hill & Chow, 2002; Mun et al., 2018). Specifically, we will test substance use to assess whether the results remain the same. Testing these hypotheses is not merely an academic exercise; its findings could contribute to developing more attuned targets for prevention programs, such as targeting youth’s self-regulation in resource-poor environments.

## Methods

### Participants

The present study utilized longitudinal data from the Early Steps Multisite (ESM) study, a randomized controlled trial investigating the prevention of children’s early emerging behavioral problems (Dishion et al., 2008). A detailed description of the Family Check-Up intervention used in the ESM study is available elsewhere (Dishion & Kavanagh, 2003; Dishion & Stormshak, 2007), but this report does not focus on the intervention.

The sample included 731 children and their primary caregivers recruited between 2002 and 2003. Over 96% of the primary caregivers at the initial assessment were biological mothers; in all other cases, they were non-maternal custodial caregivers, such as biological fathers. Participants were recruited from Women, Infants, and Children (WIC) Nutritional Supplement Centers in the metropolitan areas of Pittsburgh (Pennsylvania), Eugene (Oregon), and Charlottesville (Virginia). Families were eligible to participate if they had a child between 24 and 35 months (*M* = 29.9 months, SD = 3.2) and met risk criteria in at least two of three risk domains for future behavioral problems. Specifically, risk criteria for recruitment were defined at one standard deviation or above the normative range on several screening measures within these three domains: (a) child behavior (conduct problems, high-conflict relationships with adults); (b) family problems (maternal depression, daily parenting challenges, substance use problems, teen parent status); and (c) socio-demographic risk (no more than 2 years post-high school education and low family income). Children who met criteria based on only family problems and socio-demographic risk were also required to have above-normative levels of externalizing problems to ensure significant levels of problem behavior.

Of the 1,666 parents approached at WIC sites across the three study sites and who had children in the appropriate age range, 879 families met the eligibility requirements; of these, 731 agreed to participate. No differences in family problems, socio-demographic risk, or problem behavior appeared between those who agreed to participate and those who did not. Of the 731 families (49% female children), 272 (37%) were in Pittsburgh, 271 (37%) in the Eugene site, and 188 (26%) in Charlottesville. During the screening period, more than two-thirds of those families enrolled had an annual income of less than $20,000; the average number of family members per household was 4.5 (SD = 1.63). Forty-one percent of the sample had a high school diploma or General Educational Development equivalency, and 32% had 1 to 2 years of post-high school training.

### Procedure

Assessments took place during home visits. Families (i.e., primary caregivers) received compensation for their effort and time. For the current study, we focus on child data collected from assessments occurring at youth aged 4.5, 7.5, 9.5, 10.5, 14, and 16. Attrition analysis indicated missing data were unrelated to the study variables, suggesting that the missing data in our study adheres to the missing completely at random assumption.

### Measures

#### Pubertal timing at age 10.5.

At youth age of 10.5, children reported their pubertal status using the measure developed by Petersen et al. (1988). Pubertal characteristics measured included height, pubic hair, and skin changes for boys and girls; facial hair growth and voice change for boys only; and breast development and menarche for girls only. For each characteristic, respondents rated whether development had not yet started (1), had barely begun (2), was underway (3), or was completed (4) (*α* = 0.61). An overall pubertal development score was computed by summing the five items to obtain a total score. The Pubertal Development Scale is a widely-used self-report measure of physical development for youth under the age of 16, and it has been shown to correlate with measures of pubertal development derived from physical examination (Icenogle et al., 2017). Due to the sensitive nature of these items, we obtained verbal permission from the primary caregiver before administration to target children.

#### Effortful control at age 14.

We focused on effortful control as an indicator of self-regulation because it is a broad term that includes different dimensions (i.e., activation control, attention control, inhibitory control). The Early Adolescent Temperament Questionnaire (EATQ-R) was used to measure effortful control (Ellis & Rothbart, 2001). It includes three dimensions of regulatory temperament: activation control (i.e., capacity to perform actions when there exists a strong tendency to avoid actions; “If you have a hard assignment to do, you get started right away,” *α* = .70), inhibitory control (i.e., capacity to suppress inappropriate responses; “When someone tells you to stop doing something, it is easy for you to stop,” *α* = .54), and attention control (i.e., capacity to focus and shift attention; “You are good at keeping track of several different things that are happening around you,” *α* = .64). Adolescents rated the frequency with which each item applied to them over the past six months on a 5-point Likert-type scale (1 = Almost never or never to 5 = Almost always or always). Together the three dimensions of the EATQ-R form indicators of the latent construct of Effortful Control.

#### Annual household income.

Annual household income was reported by parents using an ordinal scale (1 = less than $4,999; 2 = $5,000–$9,999; 3 = $10,000–$14,999; 4 = $15,000–$19,999; 5 = $20,000–$24,999; 6 = $25,000–$29,999; 6 = $30,000–$39,999; 7 = $40,000–$49,999; 8 = $50,000–$59,999; 9 = $60,000–$69,999; 10 = $70,000-$79,999; 11 = $80,000–$89,999; 12 = $90,000 or more) at youth age 4 (2005, 2006, and 2007) and 7 (2008, 2009, and 2010). In total 77.9% of the families at child age 4.5 and 62.1% at child age 7.5 had an annual family income of less than $30,000 a year, including child support and other financial aid. Household income is imprecise regarding the specification of the family- and individual-level exposure to scarcity because of wide variations in household size and cost of living. Therefore, we used an Income-to-Needs (ITN) index, which accounts for household size and income relative to the federal poverty line (e.g., Kim et al., 2019; Ursache & Noble, 2016). Based on the measurement for annual household income at child age 7.5 and the ITN ratio calculation of related year, we deemed half of the sample to be “low-ITN” (50.7% of the sample; ITN <1), 40.8% “medium-ITN” (ITN <2), and 8.5% “high-ITN” (ITN ≥2) (c.f. Deater-Deckard et al., 2019). If the measurement of household income was missing, ITN was calculated based on household income assessed at child age 4.5. Our sample consisted of many children who grew up in low-ITN families, and the high-ITN group was relatively small, so we combined the groups, including medium and high-ITN families (total *N* = 637).

#### Sexual activity at age 16.

Sexual activity was assessed by calculating the mean of three separate items (i.e., During your life, with how many people did you have oral sex; During your life, with how many people did you have vaginal sex?; During your life, with how many people did you have anal sex?). Items were scaled on a continuous scale, ranging from 0 to 6 (six or more people). The number of sexual partners is a valid indicator of sexual activity (Martinez & Abma, 2020)

#### Substance use at age 16.

Participants were asked whether they had ever used cigarettes (vapor), beer, wine, liquor, (synthetic) cannabis, LSD, prescription drugs, steroids, inhalants (e.g., glue), heroin, or cocaine. A sum score of the dummy-coded variables for the different substances was calculated as a general substance use index (e.g., Spoth et al., 2001).

### Covariates

Children’s gender (49.3% females), age (at first measurement: Mean = 49.50 months, SD = 3.17), and race were included as covariates in the model. In total, 49.6% of respondents were White, 28.5% were Black-African American, and 21.9% were other races, including Native American (1.3%), bi-racial (13.2%), and other races (7.4%). As data were part of a randomized controlled trial to test the effectiveness of the Family Check-Up, we controlled for treatment group assignment (i.e., control group [49.7%] versus intervention group [50.3%]). Finally, to control for earlier levels of self-regulation, we included a measure of parent-reported Effortful Control (activation control *α* = .69; inhibition control *α* = .85; attention control *α* = .78: Rothbart et al., 2001) at age 7.5.

### Measurement model for effortful control

We tested the measurement model for effortful control assessed at ages 7.5 and 14 by means of automated invariance testing in Mplus (Muthén & Muthén, 1998–2015). Automated invariance testing allows for the test of the configural, metric, and scalar invariance. The fit measures for each of the three measurement models (configural, metric, scalar) were very good with RMSEA’s lower than .05, CFI’s and TLI’s higher than .95 (Kline, 2015; Steiger, 1990). Each of the models was not rejected as indicated by insignificant chi-squares, showing support for invariance.

### Data analysis

Using Mplus version 7.4, we employed structural equation modeling to test the hypothesized model for the two income groups (i.e., low-ITN, medium/high-ITN). As sexual activity at age 16 was skewed with a preponderance of zeros, we used a zero-inflated Poisson distribution (Muthén & Muthén, 1998–2015). The default estimation method is MLR which estimates with standard errors and a chi-square test statistic, both robust to non-normality and non-independence of observations when used with type = complex. The MLR standard errors are computed using a sandwich estimator. Estimates for both groups were obtained with the KNOWNCLASS option for grouping (Muthén & Muthén, 1998–2015). Subsequently, using the Model Constraints methods, new latent variables were constructed to test differences between the two groups and whether children’s effortful control during middle childhood (i.e., age 14) would mediate the effects of pubertal timing on sexual activity (i.e., indirect effects) (e.g., Muthén & Muthén, 1998–2015).

### Transparency and openness

All data are available at [https://osf.io/nzws2/]. All analysis code and research materials can be obtained from the first author. The study’s design and its analysis were not pre-registered.

## Results

First, we calculated descriptive statistics. Around 75% of the sample at age 16 reported never having sex. Next, bivariate correlations on the total sample were calculated to examine initial associations (see Table 1). Pubertal timing was higher in female participants and in participants of another race than white. Income-to-Needs was lower in families of another race than white. Substance use was higher in participants from families of another race than white.

Subsequently, we used path analyses to test the extent to which pubertal timing was related to effortful control, which in turn was hypothesized to predict rates of sexual activity in the two ITN groups. An estimator for count data does not provide regular fit measures such as the CFI, TLI, or RMSEA. The BIC can be used to compare the relative fit of different models to obtain some idea of the overall fit (Burnham & Anderson, 2004). We have compared our model to a model in which all means and variances were freed and to a model in which all means and variances were constrained to zero. In each of these models, the BIC was worse than our baseline model (12.957,749). Factor loadings of the latent constructs of effortful control for age 7.5 and age 14 ranged from 0.66 to 0.87.

**Figure 1.**
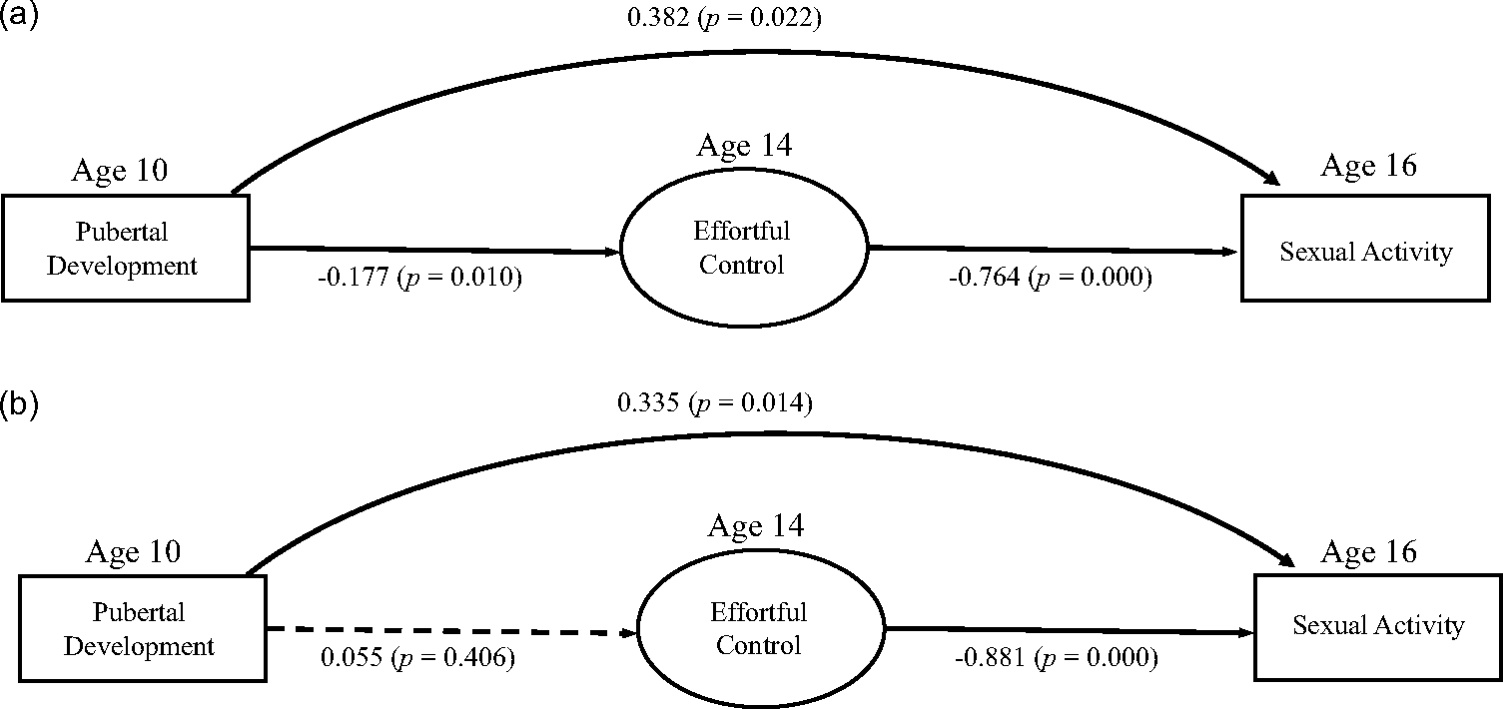
(a) Low-ITN group; (b) High-ITN group.

Figure 1a shows the results for the low-ITN group. Regarding covariates, sexual activity was associated with gender, such that female participants were less active than male participants (*B* = − .345, *p* = .023). The autoregressive pathway of effortful control between age 7.5 and age 14 was significant with a standardized estimate of 0.319, *p* = .000). Pubertal timing at age 10 was associated with being female (*r* = .143, *p* = .000) and with having another race than white (*r* = 135, *p* = .002). Concerning the main study variables, pubertal timing was a significant negative predictor of effortful control (*B* = −0.177, *p* = .010). Effortful control was a significant negative predictor of sexual activity (*B* = −0.764, *p* = .000). Pubertal timing was also directly associated with sexual activity (*B* = 0.382, *p* = .022).

Figure 1b shows the results for the medium/high-ITN group. Regarding covariates, sexual activity was associated with gender, such that female participants were less active than male participants (*B* = −.302, *p* = .032). Moreover, the autoregressive pathway of effortful control between age 7.5 and age 14 was significant with a standardized estimate of 0.324, = .000). Pubertal timing at age 10.5 was associated with being female (*r* = .143, *p* = .001) and with having another race than white (*r* = .135, *p* = .002). In this group, pubertal timing was not associated with effortful control (*B* = 0.055, *p* = .406), effortful control did, however, predict sexual activity (*B* = −0.881, *p* = .000). Pubertal timing was also a predictor of sexual activity in the model (*B* = 0.335, *p* = .014).

The newly constructed latent variables to test differences and indirect effects showed support for one significant indirect effect linking pubertal timing with subsequent effortful control and sexual activity in the low-ITN group (unstandardized estimate = .037, *p* = .034). The link between pubertal timing and effortful control significantly differed between the two groups (unstandardized estimate = −.060, *p* = .009). The link between effortful control and sexual activity was not different for the two groups (unstandardized estimate = −.270, *p* = .714). The indirect effect in the low-ITN group was also significantly different from the indirect effect in the medium/high-ITN group (*b* = .052, *p* = .023).

### Analyses of substance use as outcome

Following the idea that earlier pubertal timing and related underdeveloped self-regulation (Brooks-Gunn et al., 1985) underlie adolescents’ engagement in risk behavior (Westling et al., 2012), we conducted the analyses again with substance use at age 16 as an outcome to test the robustness of our findings. Findings were similar with pubertal timing being a relevant predictor of effortful control (standardized estimate −0.150 (*p* = .028), which in turn, was a relevant predictor of substance use (standardized estimate −0.777, *p* = .000), but only in the low-ITN group. The link between effortful control and pubertal timing was also significantly different in both groups (unstandardized estimate −0.058 95% CI [−0.108, −0.008]). Substance use was not directly associated with pubertal timing. However, the indirect effect of pubertal timing on substance use via effortful control was not significant (unstandardized estimate 0.017 95% CI [−0.004, 0.039]).

## Discussion

Deater-Deckard et al. (2019) found that pubertal timing and neurocognitive self-regulation operate in distinct ways in resource-poor environments (i.e., low-ITN), compared with environments containing adequate resources (i.e., medium- and high-ITN). The current work replicated those findings with a different paradigm and extended this body of research by taking a more behavioral approach within a longitudinal design. Specifically, we found that in the low-ITN group, but not in the medium/high-ITN group, higher scores on pubertal timing at age 10 were linked to lower levels of effortful control at age 14 (controlling for earlier effortful control at age 7.5), and less effortful control was associated with more sexual activity at age 16. These findings were partially replicated by using substance use at age 16 as an alternative outcome, although we did not find support for an indirect effect with this outcome.

From a theoretical perspective, this study established a potential mechanism explaining *why* the link between pubertal timing and risk behavior operates differently in youth who grow up in resource-poor environments compared to those growing up in environments with at least adequate resources. Specifically, different evolutionary models -including the LHT- suggest that growing up in an environment characterized by low resources may trigger accelerations in pubertal maturation, with the evolutionary goal of increasing reproductive fitness quickly (Ellis et al., 2012). However, our findings only partially supported the underlying idea of LHT; they did not show evidence for the r/K-selection theory because there was no association between the level of pubertal timing and ITN. However, our study did show that in children from low-ITN families, early pubertal timing had different consequences on regulation and sexual activity (and substance use). Accordingly, adolescents’ sexual activity from low-ITN families is more likely to result from reduced levels of effortful control than children from medium- or high-ITN families. Future studies should further identify the consequences of effortful control development in low-resource environments. As the extra analyses showed that effortful control in resource-poor environments also might affect substance use (as an indicator of other risk behavior than sexual activity), our findings likely expand to other behaviors (e.g., aggression) or mental states (e.g., depressive symptoms).

The model we tested has potential implications for designing preventive interventions for high-risk youth and suggests new research directions that have not been forthcoming from other perspectives. For instance, it indicates that schools in low-resource environments may need a different approach to preventing risky behavior than those with adequate or high resources. Specifically, early puberty may be more likely to affect the magnitude of association between effortful control and subsequent high-risk behavior in low-resource settings. Therefore, prevention programs in these contexts should identify and implement preventive interventions for children with low levels of effortful control at earlier ages than in environments or neighborhoods with adequate resources. A review by Fryer and Katz (2013) showed that school interventions were more effective than neighborhood interventions in improving children’s long-term outcomes in poor-resource families. While higher-quality neighborhoods *did* improve family safety, adult well-being and health, and girls’ mental health, it did not have detectable impacts on youth human capital, labor market outcomes, or risky behaviors. In contrast, higher-quality schools *did* improve children’s academic achievement and had longer-term positive impacts of increasing educational attainment and earnings and reducing incarceration and teen pregnancy (Fryer & Katz, 2013).

Future studies also should concentrate on mechanisms used by children (and their parents) who grow up in resourceful families to counteract the links between puberty, effortful control, and sexual activity. For instance, it may be that children who grow up in high-resource environments have experienced more effective parenting (or more specific parenting practices aimed at sex education), protecting these children from the adverse consequences of early pubertal timing. Similarly, studies have shown that parents who engage in behaviors to prevent smoking during early adolescence have children who are less likely to initiate smoking (Hiemstra et al., 2012).

## Strengths and limitations

This study has some strengths and limitations. First, the measurements in this study are strong, with valid measures for effortful control. To assess social adversity, we used Income-to-Needs as an indicator, a more accurate measure of socioeconomic status than family income (Ursache & Noble, 2016). The long-term longitudinal character of the study with data covering children ages 4.5–16 allowed us to test the developmental process articulated by the LHT. Another strength of the study is the sample, which consisted of a substantial low-ITN group, often under-sampled in social sciences. A final strength pertains to the sensitivity analyses that reproduced similar findings with substance use as an outcome measure. These analyses establish the robustness of the study findings.

As in every study, there are also limitations. First, the literature on the LHT has grown rapidly in recent years with increasingly more psychological research focused on humans (Nettle & Frankenhuis, 2019). Most research concentrates on adverse circumstances and how such conditions negatively affect behavior. However, this might be considered an adaptive response to those circumstances. Lower effortful control may be influenced by pubertal timing, but effortful control is neither adaptive nor maladaptive. Whether lower effortful control is adaptive or maladaptive depends on the environment and the specific aspect of effortful control (i.e., inhibitory control, attentional control, activation control) (Fenneman et al., 2022). Future studies should examine whether there are other consequences of changes in effortful control due to lower income that are usually overseen. For instance, an adaptive response to growing up in a low-resource environment could be a behavior characterized by more creativity or willpower to extricate oneself from poverty. Finally, here low-ITN was considered as a form of a harsh environment. However, there may very well be low-ITN families in which the environment – apart from ITN – is supportive and not considered harsh.

Second, even though the sample size of our study was substantial, future studies should preferably use larger sample sizes to replicate the current pattern of results as sample sizes were relatively small. Future studies could also benefit from more diverse samples to test whether the effects shown in our study would hold in other more economically diverse samples, which may increase the generalizability of findings. In addition, to test the robustness of the findings, comparable hypotheses should be tested with similar theoretical constructs using different measures. This issue also pertains to some of the measures’ psychometric qualities. For instance, although income-to-need represents a step forward in assessing a family’s economic status relative to using more traditional measures of economic status that do not consider the family size, a more objective measure of income would be more reliable and less subject to inaccuracy and social desirability. Moreover, the reliability of the subdimensions of effortful control, particularly inhibitory control, was limited, which questions the validity of these constructs. Different perspectives on effortful control (for instance, using different reporters) improve the measurement. In addition, using effortful control as a mechanism underlying the link between early puberty and later sexual activity may also provide a relatively limited picture. The literature has shown that increased risky behavior during adolescence results from the interplay between control and impulsivity and sensation seeking (Casey et al., 2008; Ernst et al., 2005; Steinberg, 2005). Future studies would benefit from including impulsivity or sensation-seeking measures. Adding one or more of these measures would provide a more complete picture of what happens during adolescence. Finally, although the Pubertal Development Scale is a widely-used self-report measure of physical development for youth under the age of 16, and it has been shown to correlate with measures of pubertal development derived from physical examination (Icenogle et al., 2017), a more objective measure in addition to the self-report instrument would be more optimal and reliable. The dependent variable of sexual activity would be strengthened by also incorporating age at first sex, using protection, other promiscuous sexual behaviors, and the number of offspring participants were biologically responsible for by early adulthood (Dishion et al., 2012). Finally, it would be interesting to test how much sexual activity (and substance use) results from endorsed norms and attitudes in the peer group (Dishion et al., 2012). Adolescents with less effortful control in the low-resource group may be selecting and susceptible to peer influences about promoting sexual activity and drug use. In addition, from an LHT perspective, it could be argued that it is beneficial to be around peers who are likely to show similar accelerations in pubertal development and subsequent behavior.

In sum, this study found that the link between pubertal timing, self-regulation, and sexual activity operates in distinct ways in resource-poor environments. The findings partially support evolutionary models such as the LHT, which posits that stable differences in environments lead to variations in biologically influenced reproduction systems and behavior in ways that increase the likelihood of producing viable offspring within that ecological niche (Ellis et al., 2012). Ideally, prevention programs to address these pathways could be delivered before the onset of adolescence to prepare youth for greater physical and psychological mobility and the increased risk of life-changing consequences for poor decision-making during this developmental period, such as sexual risk-taking and substance use.

## Figures and Tables

**Table 1.** Bivariate correlations between the study variables

	Pubertal timing	Activation control	Inhibitory control	Attention control	Sexual activity	Substance use	ITN	Gender	Age	Race	*N*, Means (Standard Deviations) or proportions
Pubertal timing	-										*N* = 561, *M* = 9.37 (SD = 2.20)
Activation control	−0.07	-									*N* = 544, *M* = 3.29 (SD = 0.75)
Inhibitory control	−0.02	0.51**	-								*N* = 547, *M* = 3.89 (SD = 0.64)
Attention control	−0.05	0.59**	0.52**	-							*N* = 548, *M* = 3.50 (SD = 0.63)
Sexual activity	0.07	−0.11*	−0.06	−0.06	-						*N* = 714, *M* = 0.29 (SD = 0.72)
Substance use	0.03	−0.14*	−0.05	−0.08	0.44**	-					*N* = 565, *M* = 1.65 (SD = 2.30)
ITN	−0.01	0.02	−0.02	0.05	0.00	0.08	-				*N* = 637, *M* = 1.10 (SD = 0.63)
Gender	0.14**	0.00	0.00	−0.07	−0.03	0.06	−0.01	-			*N* = 714, 50.7% (males)
Age	0.06	−0.03	−0.01	0.03	0.02	−0.01	0.03	0.03	-		*N* = 714, *M* = 29.99 (SD = 3.17)
Race	0.13**	−0.00	−0.09*	−0.05	0.00	−0.14**	−0.16**	0.07	0.06	-	*N* = 714, 49.6% (white)
Group	0.03	−0.08	−0.04	0.01	0.00	0.01	−0.04	0.01	−0.08*	0.01	*N* = 714, 49.7% (control)

Note.

* *p* < 0.05,

** *p* < 0.01;

ITN = Income-to-Needs; Gender was coded 0 = male, 1 = female; race was coded White (0) vs. other (1), age was measured in months at first measurement.
